# Selection bias in general practice research: analysis in a cohort of pregnant Danish women

**DOI:** 10.1080/02813432.2020.1847827

**Published:** 2020-11-26

**Authors:** Ruth K. Ertmann, Dagny R. Nicolaisdottir, Jakob Kragstrup, Volkert Siersma, Gritt Overbeck, Philip Wilson, Melissa C. Lutterodt

**Affiliations:** a The Research Unit for General Practice and Section of General Practice, Department of Public Health, University of Copenhagen, Copenhagen, Denmark;; b Centre for Rural Health, University of Aberdeen, Aberdeen, Scotland

**Keywords:** General practices, selection, representativity, participating patients, pregnancy

## Abstract

**Objective:**

The aim of the present study was to examine selection in a general practice-based pregnancy cohort.

**Design:**

Survey linked to administrative register data.

**Setting and subjects:**

In spring 2015, GPs were recruited from two Danish regions. They were asked to invite all pregnant women in their practice who had their first prenatal care visit before 15 August 2016 to participate in the survey.

**Outcome measures:**

The characteristics of GPs and the pregnant women were compared at each step in the recruitment process – the GP’s invitation, their agreement to participate, actual GP participation, and the women’s participation – with an uncertainty coefficient to quantify the step where the largest selection occurs.

**Results:**

Significant differences were found between participating and non-participating practices with regards to practice characteristics such as the number of patients registered with the practice, the age and sex of doctors, and the type of practice. Despite these differences, the characteristics of the eligible patients differed little between participating and non-participating practices. In participating practices significant differences were, however, observed between recruited and non-recruited patients.

**Conclusion:**

The skewed selection of patients was mainly caused by a high number of non-participants within practices that actively took part in the study. We recommend that a focus on the sampling within participating practices be the most important factor in representative sampling of patient populations in general practice.

**Key points**
Selection among general practitioners (GPs) is often unavoidable in practice-based studies, and we found significant differences between participating and non-participating practices. These include practice characteristics such as the number of GPs, the number of patients registered with the GP practice, as well as the sex and age of the GPs.
•Despite this, only small differences in the characteristics of the eligible patients were observed between participating and non-participating practices.
•In participating practices, however, significant differences were observed between recruited and non-recruited patients.
•Comprehensive sampling within participating practices may be the best way to generate representative samples of patients.

## Introduction

Population-based research in primary care generally depends on gaining access to primary care settings and recruiting patients with a degree of diversity representative of the population. General practitioner (GP) participation is a crucial component. Many research projects in general practice are based on self-selection among GPs [1], raising questions about possible selection bias. All GPs may be invited to participate in a study, but not all decide to take part. For simple questionnaires aimed at GPs, response rates just above 50% are common [2–5]. More complex projects, which involve the inclusion of patients and extra clinical work, often have lower participation rates [6]. Various barriers to GP recruitment in research have been identified, including practical and organisational factors, such as competing time commitments and a lack of reimbursement [7], as well as personal factors [8].

Consecutive sampling of attending patients is widely used, given the simplicity and ease of implementation. The implications of using such an approach has, however, received scant attention. The representativeness of a visit-based sample was compared with the population of patients seen during the same year, and it was found that sampling of consecutive attenders typically underrepresents low users of the service [9]. Other studies have shown lower levels of participation among less privileged groups of society [10]: people with low income or low educational levels have typically been underrepresented in cohort studies in Western society [11]. Participation rates have also varied according to, for example, age and sex [11]. Sampling bias may, therefore, arise in various ways: GPs may decide not to participate, they may not invite some patients to participate, or patients may decide themselves not to accept the invitation [12,13]. Knowing the extent of these mechanisms would be valuable in developing strategies to obtain representative samples.

Studies of non-participation, aiming to compare data for participating and non-participating GPs and patients, must rely on population data produced independently of the study, in particular administrative register data. In Denmark, such data are available to researchers on an anonymised basis in national registers.

The aim of the present study was to examine selection in a general practice-based pregnancy cohort. We studied the differences in a range of practice characteristics and characteristics of the pregnant women at each step in the recruitment process. The steps in which the largest differences occur are the most critical in avoiding selection bias, and we discuss some implications of our results for study design.

## Material and methods

### Setting

The healthcare system in Denmark is tax-funded, and care is free of charge for the patient. The majority of Danes are registered with a GP who functions as gatekeeper to specialist secondary care. Citizens are entitled to a regular GP of their own choosing and thereby become registered with a practice. Some practices are small and single handed, while other practices comprise 2–6 GPs, who own the clinic jointly and share a larger number of patients.

By law, a minimum of three prenatal care visits are offered by the GP, at pregnancy weeks 6–10, 25, and 32. A fourth postnatal care visit is conducted 8 weeks after delivery. The first visit is attended by almost all pregnant women wanting to keep their pregnancy and precedes other pregnancy-related contacts in the healthcare system. In this consultation, a thorough and structured record is established (the Pregnancy Health Record), which is then sent to midwives and the hospital department.

### Recruitment to the study

The present report is based on the recruitment process for a cohort study of pregnant women recruited in general practice at the first prenatal care visit [14]. This study aimed to investigate the physical and mental well-being of the women during their pregnancy and postpartum.

All GPs working in the Capital Region of Denmark and Region Zealand, two of the five Danish administrative regions, were eligible to participate in the study. In spring 2015, a subgroup of these practices was selected and invited to participate and recruit pregnant women to the study. A systematic procedure was used for the selection: first, all practices were allocated to geographically defined subgroups using municipalities and postal codes. These subgroups were randomly ranked and the practices in these subgroups were then contacted in the order of the ranking. The initial contact was a telephone call from the principal investigator (RE) to the GPs, and if the GP indicated interest, this was followed up by an email with detailed information and, on some occasions, a visit to the practice.

GPs who accepted participation (before 30 June 2015) were asked to invite all women booking an appointment for a first prenatal care visit in the recruitment period until 15 August 2016. GPs were offered a fee for each pregnant woman recruited to the study, an amount corresponding to reimbursement for one normal consultation for each woman.

During the study period, there was frequent communication between the principal investigator (RE) and the participating practices about recruitment, including e-mails about the progression of the study.

### Identification of the source population

To identify selection differences for the purpose of the present study, data were obtained from the Danish national registers [15]. Those registers are based upon a 10-digit civil registration number assigned to all individuals in Denmark at birth or upon immigration to provide a unique identifier. Based on the civil registration number, Statistics Denmark provided an anonymised linkage between data on all pregnant women who were listed with the participating GP practices and had attended the first prenatal care visit (coded by the GP for remuneration purpose). Using the specific code (8110), it was possible to extract data on all women attending the first prenatal care visit in each practice during the recruitment period. Approval from the Danish Data Protection Agency was obtained (Journal 2014-41-3018). According to Danish law, observational studies and studies based entirely on data collected from registers do not need approval from a scientific ethics committee.

### Variables

The following variables were obtained from the registers about the pregnant women: age in years (<25, 26–30, 31–35, >35), marital status (married, cohabiting, single), country of origin (Denmark, other), years of completed education, (<3 years, 3–5 years, >5 years), occupation (employed, unemployed, student, other), household income (<39,999 EURO, 40,000–79,999 EURO, 80,000–119,999 EURO, ≥120,000 EURO, missing), whether she had previously given birth (yes, no), frequency of consultations (1–5, 6–10, >10 in the previous calendar year), use of medication for diseases of the central nervous system, e.g. medicines for depression, anxiety, schizophrenia, epilepsy, pain (ACT classification code N) in the previous year.

The following variables were obtained about the practices: number of patients (<1800, 1800–2000, >2000), type of practice (single-handed, group practice, other), geography (city, country), average age of the doctors (≤50, 51–60, >60), the sex of the doctors in the practice (male, female, both sexes).

### Statistical analysis

Differences were studied in practice characteristics between practices wishing to participate (*accepted participation*) versus practices not wishing to participate (*declined participation*), and differences between practices that actually recruited patients to the study and practices that agreed to participate, but did not recruit patients. Selective recruitment was studied by means of administrative data based on practice and patient characteristics. Finally, the characteristics of the recruited patients were compared to patients that were not recruited.

To compare the strength of the various selection effects for a certain characteristic, uncertainty coefficients [16–18] are calculated. This coefficient is the percentage reduction of the variation of the characteristic (measured as entropy) due to a selection effect. As the selection effects are binary indicators, e.g. participating practice and non-participating practice, the variation that remains after removing a selection effect is calculated by pooling the two within-group variations. The value of the coefficient tends to decrease to 0 as the number of categories of the characteristic increases, so there is no benchmark value indicating a particularly strong effect. However, higher values of the uncertainty coefficient indicate stronger selection effects, and coefficients for the same characteristic are compared so as to determine the strongest selection effect for that characteristic. A two-stage non-parametric bootstrap was used to obtain 95% confidence intervals for these coefficients, accounting for clustering of women in practices. The statistical analyses were performed in SAS version 9.4 (SAS Institute, Cary, NC) and R version 3.5.1.

## Results

**Figure 1.**
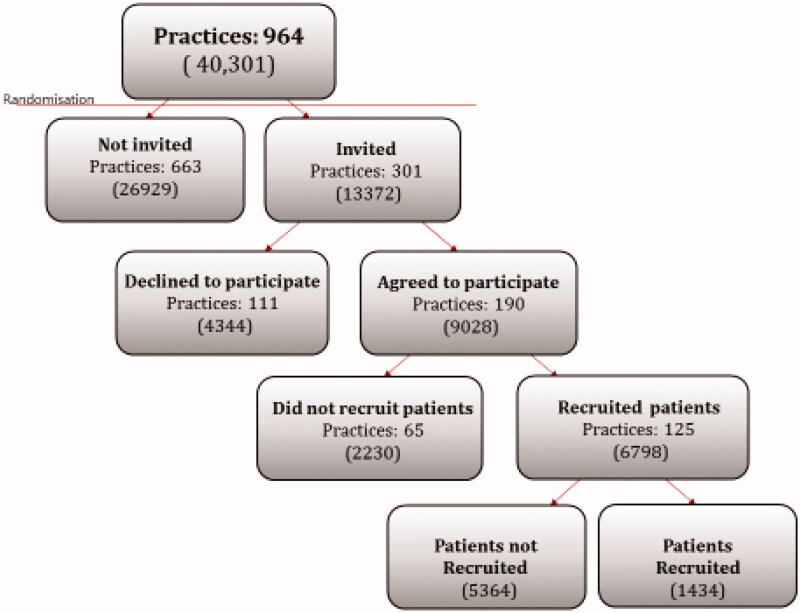
Inclusion of GP practices and pregnant women (in parenthesis) in the Capital Region of Denmark and Region Zealand.

The invitation, participation, and recruitment processes are shown in Figure 1. We invited 305 out of 1561 general practices in the Capital Region of Denmark and Region Zealand, following the systematic randomisation procedure. A total of 190 practices (62% of those invited) agreed to participate, but only 125 (41% of those invited) recruited one or more pregnant women during the study period. These active practices recruited an average of 12 women (range 1–84) and only 1508 (17%) were recruited to the study from the 9028 eligible pregnant women who attended the first prenatal care visit at a practice that had agreed to participate. For four individual women in the study, we were not able to determine their GP. During the study some women moved, some could not be traced because of an incorrect or missing civil registration number, and others had a spontaneous abortion; this left 1434 out of 1508 women to participate in the study.

Table 1 shows the characteristics of the practices at each step in the selection process. As seen from the uncertainty coefficients, the most pronounced differences in practice characteristics (number of patients on the list, type of practice, age and sex of doctors) were between practices that recruited women and those that did not recruit women. Geographic location of the practice was the only factor for which the difference was largest between those practices that were invited and those not invited.

**Table 1.** Differences in practice characteristics between practices that were invited into the study versus practices that were not invited into the study, practices that accepted participation into the study versus practices that declined to participate among those invited, and practices that recruited women into the study versus those that did not recruit women into the study among those which had agreed to participate.

		Practice invited							
		Practice accepted participation				Practice did not accept participation		Practice not invited	
		Practice recruiting		Practice not recruiting					
		*N* = 125	12.97%	*N* = 65	6.74%	*N* = 111	11.51%	*N* = 663	68.78%
Number of patients			3.23 (0.26–9.63)		2.13 (0.19–6.01)		0.71 (0.06–1.96)		
	<2000	36	28.80	31	47.69	53	47.75	328	49.47
	≥2000	82	65.60	29	44.62	43	38.74	277	41.78
	Missing	7	5.60	5	7.69	15	13.51	58	8.75
Type of practice			3.67 (0.92–9.24)		2.96 (0.86–6.96)		1.73 (0.74–3.35)		
	Single	42	33.60	36	55.38	71	63.96	430	64.86
	Group	59	47.20	15	23.08	27	24.32	180	27.15
	Other	24	19.20	14	21.54	13	11.71	53	7.99
Geographic location			0.00 (0.00–2.23)		2.14 (0.22–6.14)		4.86 (2.61–7.79)		
	City	56	44.80	28	43.08	68	61.26	523	78.88
	Country	60	48.00	31	47.69	36	32.43	140	21.12
	Missing	9	7.20	6	9.23	7	6.31	0	0.00
Average age of doctors			2.47 (0.38–7.94)		0.02 (0.01–1.65)		0.20 (0.02–1.01)		
	≤50 years	47	37.60	14	21.54	33	29.73	190	28.66
	51–60 years	49	39.20	25	38.46	39	35.14	237	35.75
	>60 years	22	17.60	21	32.31	25	22.52	186	28.05
	Missing	7	5.60	5	7.69	14	12.61	50	7.54
Sex of doctors			6.22 (2.71–12.33)		2.44 (0.84–6.04)		0.45 (0.11–1.44)		
	Male	23	18.40	32	49.23	41	36.94	238	35.90
	Female	36	28.80	14	21.54	35	31.53	222	33.48
	Both sexes	59	47.20	14	21.54	21	18.92	153	23.08
	Missing	7	5.60	5	7.69	14	12.61	50	7.54

The boxes show the uncertainty coefficient in % (95% confidence interval in brackets), which quantifies the difference in distribution and thereby the relative strength of the selection effect in each step for each characteristic of the women. *The uncertainty coefficient builds on Goodman and Kruskal’s classic review of association measures* [18].

Table 2 shows characteristics of all women who had a first prenatal care visit in the study period for each step in the selection process. Some effect of the sampling process was observed at all steps, but the uncertainty coefficients indicate that the most pronounced differences in socio-demographic characteristics were found between included and non-included women within practices that recruited patients. Patients that were included in the active practices were less likely to live alone and more likely to be born in Denmark, well educated, employed, have a higher household income, have other children, and have fewer contacts with the GP per year. However, for the women’s age and their use of prescription medicines for central nervous system (ATC-code N), the largest differences were seen at the initial invitation stage, i.e. between invited and non-invited practices in our study.

**Table 2.** Differences in characteristics of the pregnant women between practices that were invited into the study versus practices that were not invited into the study, practices that accepted participation in the study versus practices that declined to participate among those invited and practices that recruited women into the study versus those that did not recruit women into the study among those which had agreed to participate.

		Practice invited								Practice not invited	
		Practice accepted participation						Practice did not accept participation			
		Practice recruiting				Practice not recruiting					
		Women included		Women not included							
		*N* = 1434	3.56%	*N* = 5364	13.31%	*N* = 2230	5.53%	*N* = 4344	10.78%	*N* = 26929	66.82%
Age			0.06 (0.01–0.22)		0.03 (0.01–0.19)		0.10 (0.02–0.32)		0.25 (0.13–0.41)		
	<25 years	262	18.27	1141	21.27	447	20.04	728	16.76	3991	14.82
	26–30 years	540	37.66	1872	34.90	744	33.36	1628	37.48	9138	33.93
	31–35 years	416	29.01	1522	28.37	667	29.91	1272	29.28	8582	31.87
	>35 years	216	15.06	829	15.45	372	16.68	716	16.48	5218	19.38
Marital status			0.22 (0.09–0.44)		0.04 (0.00–0.20)		0.05 (0.00–0.20)		0.01 (0.00–0.04)		
	Married	563	39.26	2258	42.10	914	40.99	1675	38.56	11092	41.19
	Cohabiting	675	47.07	2178	40.60	901	40.40	1860	42.82	11310	42.00
	Living alone	196	13.67	928	17.30	415	18.61	809	18.62	4527	16.81
Country of origin			3.01 (2.15–3.93)		0.20 (0.00–1.21)		0.04 (0.00–0.32)		0.08 (0.00–0.27)		
	Denmark	1316	91.77	4051	75.52	1660	74.44	3292	75.78	20018	74.34
	Other	118	8.23	1313	24.48	570	25.56	1052	24.22	6911	25.66
Education level			1.41 (1.01–1.99)		0.09 (0.04–0.49)		0.43 (0.13–1.01)		0.58 (0.31–0.97)		
	Primary school	171	11.92	931	17.36	399	17.89	610	14.04	3051	11.33
	Educatio*n* = 3 years	583	40.66	1984	36.99	780	34.98	1317	30.32	7827	29.07
	Educatio*n* = 4 or 5 years	436	30.40	1152	21.48	473	21.21	1059	24.38	6554	24.34
	Educatio*n* > 5 years	208	14.50	676	12.60	333	14.93	821	18.90	6077	22.57
	Missing	36	2.51	621	11.58	245	10.99	537	12.36	3420	12.70
Occupation			1.10 (0.72–1.64)		0.01 (0.00–0.22)		0.03 (0.01–0.20)		0.07 (0.02–0.19)		
	Employed	1125	78.45	3667	68.36	1548	69.42	3044	70.07	19486	72.36
	Unemployed	71	4.95	541	10.09	203	9.10	347	7.99	1853	6.88
	Student	171	11.92	576	10.74	245	10.99	515	11.86	3049	11.32
	Other	67	4.67	580	10.81	234	10.49	438	10.08	2541	9.44
Household income			0.90 (0.52–1.45)		0.07 (0.01–0.35)		0.10 (0.04–0.27)		0.40 (0.23–0.60)		
	≤39,999 EUR	160	11.16	1129	21.05	479	21.48	915	21.06	4811	17.87
	40,000–79,999 EUR	468	32.64	1928	35.94	816	36.59	1505	34.65	8610	31.97
	80,000–119,000 EUR	565	39.40	1608	29.98	661	29.64	1230	28.31	7704	28.61
	≥120,000 EUR	241	16.81	690	12.86	274	12.29	691	15.91	5755	21.37
	Missing	0	0.00	9	0.17	0	0.00	3	0.07	49	0.18
Previously given birth			0.54 (0.08–1.39)		0.02 (0.00–0.11)		0.02 (0.00–0.09)		0.00 (0.00–0.01)		
	Yes	723	50.42	2176	40.57	989	44.35	1941	44.68	11785	43,76
	No	711	49.58	3188	59.43	1241	55.65	2403	55.32	15144	56,24
GP consultations per year			0.11 (0.01–0.35)		0.02 (0.00–0.21)		0.04 (0.00–0.22)		0.01 (0.00–0.06)		
	1–5 times	562	39.19	1984	36.99	860	38.57	1672	38.49	10393	38.59
	6–10 times	589	41.07	2092	39.00	838	37.58	1760	40.52	10684	39.67
	11 times or more	283	19.74	1288	24.01	532	23.86	912	20.99	5852	21.73
Use of medicine (ATC-group *N*)*			0.00 (0.00–0.07)		0.00 (0.00–0.11)		0.12 (0.01–0.36)		0.07 (0.02–0.16)		
	No	1105	77.06	4155	77.46	1724	77.31	3501	80.59	21762	80.81
	Yes	329	22.94	1209	22.54	506	22.69	843	19.41	5167	19.19

Furthermore, it shows differences in characteristic of women who were included in the study versus women who were not included in the study from the practices that actively recruited women into the study. The boxes show the uncertainty coefficient in % (95% confidence interval in brackets), which quantifies the difference in distribution and thereby the relative strength of the selection effect in each step for each characteristic of the women. *The uncertainty coefficient builds on Goodman and Kruskal’s classic review of association measures* [18].

## Discussion

### Statement of principal findings

Considerable differences were found between practices that recruited women and practices that did not recruit women. Despite these differences, the characteristics of the eligible pregnant women in these practices differed little. Within active practices, however, considerable differences were observed between women who were recruited and women who were not recruited. Selection among GPs is often unavoidable in practice-based studies, and our study shows that the selective recruitment of individuals within the practice may be most critical for the representative or balanced sampling of patient populations.

### Strength and weaknesses of the study

Demographic information on GPs and patients was studied using the Danish National Registers. It is often difficult to get information about non-participants in cohort studies [19], but this pregnancy study offered a unique opportunity to identify the source population, because almost all pregnant women in Denmark get in contact with their GP early in pregnancy, and first pregnancy consultations are registered in a national database based on reimbursement data provided by GPs. Personal characteristics and socioeconomic information are also available in the national registers enabling us to describe participants and non-participants by data of high validity, without recall bias and with low risk of misclassification compared to self-reported data [20].

Our study analysed selection at the various stages of the recruitment process in relation to both practice characteristics and the sociodemographic characteristics of the pregnant women that were available in registers. It is important to stress that the representativity of the sampled population may be different for measures not available in registers, such as the occurrence of sleep problems, physical discomfort, depressive symptoms, and other issues in pregnancy. Such measures of interest may theoretically be distributed differently in women that are recruited and women that are not recruited, irrespective of selection in the sociodemographic variables in the cohort [14]; well-being may be related to participation status even when no selection is found in sociodemographic factors, and the other way around. The problems mentioned above, however, and indeed many other health problems, do show strong associations with sociodemographic characteristics [14,21,22]. We consider our survey of selection using the available register data indicative, therefore, of a general tendency of selection at various steps of sampling in our cohort.

We observe that the selective inclusion of pregnant women within the participating practices was more important for the observed differences of most socio-demographic characteristics of the women than the selection of the GPs. Selection among doctors could be more important for the selection of other groups of patients with diseases such as hypertension, acute infections or multimorbidity; the patient lists of older doctors may, for example, comprise more patients with complex multimorbidity than lists of younger doctors. Differences between interested doctors and non-participants may also be more important if the GP has a more active role in defining the eligible patients. In our study, we were surprised to find a significant association between whether or not practices were invited with their geography and the age of their patients. We had attempted a systematic random selection of GPs, which somehow failed. Future studies should try to investigate the relative importance of selection among doctors versus selective inclusion of patients by participating doctors in other patient groups.

### Comparison to other studies of sampling in general practice

Self-selection among doctors may be difficult to avoid. Common barriers to GP participation and retention in research projects include the following: GPs having little insight into research design; concern about the misuse of patient data; scepticism about the value of the research; survey overload, lack of time, and results that are not locally relevant [23–26]. GP recruitment is time-consuming and may involve many phone calls, e-mails and visits. In one Danish study GPs were invited by letter to participate in prospective registration of patients with a respiratory tract infection; only 8.5% of the invited practices agreed to participate [27]. A study investigating barriers and facilitators to patient recruitment in primary care sent 1662 invitation letters and enrolled 55 GPs [28]. Although it is difficult and time-consuming, a personal contact with the GP seems to be more effective than asking administrative staff for permission to send the practice an e-mail containing information about a project [29,30].

GPs who agree to participate in trials do not always then recruit patients. Only 41% of the invited GPs recruited one or more pregnant women to our cohort. A Dutch study [31], investigating the effectiveness of two treatment strategies for dyspepsia, reported that 48% of the GPs recruited one or more patients. A study involving patients with menorrhagia found that 41% of GPs who agreed to participate actually recruited patients [32], while a study investigating GP and patient recruitment in a trial to determine the usefulness of brain natriuretic peptide in the diagnosis of heart failure found that 31% of the participating GPs recruited patients [33]. Higher patient recruitment rates may be promoted by establishing a relationship with GPs and clinic staff, as well as keeping regular contacts, giving clear instructions and minimising tasks for participants [29]. A study investigating the validity of a response rate of 44% obtained in a national postal study of GPs surveyed about their work with patients with alcohol abuse found some significant evidence for the presence of non-response bias, but the low response rate did not necessarily affect the validity of the data collected [34].

Recruitment may depend on a number of factors related to GPs, the topic of the investigation and patient groups. Obviously GPs, as well as patients, may be more willing to join projects that interest them. The time required to take part may also be important. First pregnancy consultations can be time consuming and this may prevent the inclusion of some patients. Concurrent studies in primary care involving pregnant women could also have lowered inclusion, but we are not aware of such studies in the study period. The number of women recruited by each of the GPs who participated in our study varied considerably, and this is similar to observations in other studies [33]. Organisational characteristics of our high-recruiter practices included: larger practices, group practices, female GPs, and practices located in rural areas or in smaller cities. Single-handed practices were over-represented among the low recruiters. A smaller number of patients may reduce the potential for recruitment and the study may thus be brought to the GP’s attention less frequently. Such effects should be studied further. Studies in the Nordic countries and New Zealand report no major GP gender differences in recruitment patterns [28,33]. A study investigating differences in medical service and the demographics of participating GPs in the five Scandinavian countries corresponds with our results: 47% of the GPs were women [35], they had a mean age of 50 and they generally shared their practice with other GPs [35]. In a study from Norway exploring the associations between GP characteristics and the quality of care for patients with type 2 diabetes, 73% of the invited practices participated and in total 55% of the GPs were male, 68% had specialist accreditation and 82% were born in Norway [36].

The characteristics associated with participation among the pregnant women were that they were born in Denmark, they were well educated and had a good income. Similarly, the Danish National Birth Cohort (DNBC), a nationwide cohort study with data from 100,000 women, showed underrepresentation of women outside the workforce, with low education levels, low income and non-Danish origin [11]. A systematic review investigating participation bias in cohort studies found an average proportion of participation to be 64% and only age, year of contact and study region were associated with participation. This leads the authors to suggest that evidence about participation and compliance should be assessed prior to funding, and local knowledge should be included in addressing the potential participants [12].

### Meaning of the study

We found significant differences between participating and non-participating practices with regard to practice characteristics such as number of GPs, number of patients registered with the GP practice and the sex and age of the GP. Only relatively small differences were, however, observed in the characteristics of the eligible patients between participating and non-participating practices. The most important differences in socio-demographic characteristics were found between those patients included and those not included in the practices that actively recruited patients. Comprehensive sampling within the participating practices may be the best way to generate representative samples of patients: the fact that some practices in our study achieved very high recruitment rates suggests that it was the GP’s invitation to participate rather than the acceptance of participation by the patient that was the crucial factor in determining level of recruitment.

Selection of a specific group of women which is not representative of all pregnant women is, however, hard to avoid. This may bias results if selection is present both in the exposure and the outcome of interest. Some of this bias may be removed by adjusting the analysis by means of observable factors with a known selection, for example, some of the factors investigated in the present paper [37]. A better approach, not necessarily possible in all studies, is to randomize the exposure or intervention; this removes confounding, notably confounding through selection.
